# X-ray detection with zinc-blende (cubic) GaN Schottky diodes

**DOI:** 10.1038/srep29535

**Published:** 2016-07-12

**Authors:** T. Gohil, J. Whale, G. Lioliou, S. V. Novikov, C. T. Foxon, A. J. Kent, A. M. Barnett

**Affiliations:** 1 Semiconductor Materials and Devices Laboratory, School of Engineering and Informatics, University of Sussex, Falmer, Brighton BN1 9QT, UK; 2 School of Physics and Astronomy, University of Nottingham, Nottingham NG7 2RD, UK

## Abstract

The room temperature X-ray responses as functions of time of two n type cubic GaN Schottky diodes (200 μm and 400 μm diameters) are reported. The current densities as functions of time for both diodes showed fast turn-on transients and increases in current density when illuminated with X-ray photons of energy up to 35 keV. The diodes were also electrically characterized: capacitance, implied depletion width and dark current measurements as functions of applied bias at room temperature are presented. At −5 V reverse bias, the capacitances of the diodes were measured to be (84.05 ± 0.01) pF and (121.67 ± 0.02) pF, respectively. At −5 V reverse bias, the dark current densities of the diodes were measured to be (347.2 ± 0.4) mA cm^−2^ and (189.0 ± 0.2) mA cm^−2^, respectively. The Schottky barrier heights of the devices (0.52 ± 0.07) eV and (0.63 ± 0.09) eV, respectively, were extracted from the forward dark current characteristics.

GaN has received extensive attention in recent years for applications such as light emitting diodes, ultraviolet detectors and more recently X-ray detectors^1^,^2^. Whilst much GaN is grown in a wurtzite (hexagonal) structure, bandgap 3.4 eV^3^, this has been found to exhibit piezoelectric polarisation effects which can result in an in-built electric field leading to the splitting of energy levels within the band-gap^4^,^5^. However, GaN grown in a zinc-blende (cubic) structure, bandgap 3.26 eV^3^, does not exhibit this behaviour^5^,^6^; this has led to increased interest in the growth and study of cubic GaN (c-GaN).

UV detection has been reported with hexagonal GaN^3^, and X-ray detection in current mode (rather than photon counting spectroscopic detection) has been demonstrated with hexagonal GaN detectors by various groups^7^,^8^,^9^,^10^,^11^. GaN’s wide bandgap, high electron mobility^11^ and thermal stability^12^ make it a material of interest for many detector applications, including future space missions to environments of high temperature and intense radiation that would be damaging to conventional semiconductor detectors.

Here, we report the photon-generated current characteristics as functions of time of two prototype n type c-GaN Schottky photodiodes at room temperature and illuminated by X-rays of energy up to 35 keV from an X-ray tube with a Mo cathode. The dark current of each diode, as a function of applied bias, was also investigated in order to determine the noise contributions to the signal response and the Schottky barrier heights of the diodes.

## Geometry of Detectors

Two silicon doped c-GaN Schottky diodes, diameters 200 μm and 400 μm, respectively, were fabricated at University of Nottingham, UK. The c-GaN layers were grown by Molecular Beam Epitaxy on (001) GaAs substrates following the procedure outlined in ref. 6. The epilayer thicknesses of the two diodes were 6 μm and 0.5 μm, respectively. Ohmic contacts were formed to the top of the epilayers using Ti (20 nm)/Al (100 nm)/Ti (5 nm)/Au (100 nm) annealed at 450 °C. The sample was glued, ohmic contact side down, to a gold-plated alumina disc using electrically conductive epoxy. The GaAs substrate was removed by a wet etch to leave only the c-GaN epilayer attached to the plated alumina disc. Schottky contacts were then formed by evaporation of 20 nm of Ni followed by 40 nm of Au through a shadow mask. Half of the areas of the Schottky contacts were covered with silver paint to enable each diode to be hand-bonded using indium wire. The two c-GaN photodiodes under test had different geometries in order to investigate the variation in dark current, capacitance and detection efficiency of c-GaN devices with area and epilayer thicknesses. Subsequent characterisation of the c-GaN photodiodes was performed at University of Sussex, UK.

## Experimental Method, Results and Discussion

**Figure 1:**
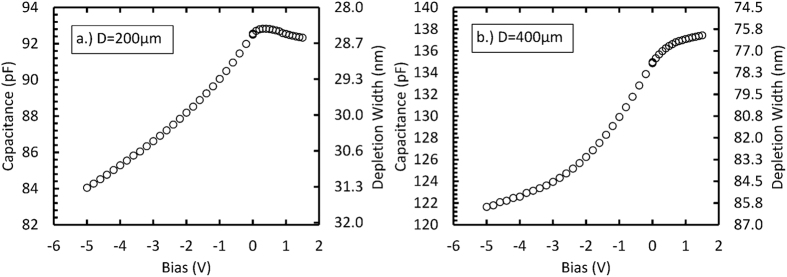
Capacitance and calculated depletion width as a function of the applied bias; (**a**) 200 μm diameter, 6 μm epilayer and (**b**) 400 μm diameter, 0.5 μm epilayer.

To determine the depletion widths of the devices, the capacitances of the packaged diodes at room temperature were measured as functions of applied bias, using an HP 4275 A Multi Frequency LCR meter with a sinusoidal waveform test signal of 60 mV and 1 MHz. The capacitance of an identical empty package was measured and subtracted from the capacitance of the packaged diodes in order to determine the capacitance of the individual diodes, Fig. 1.

As the applied reverse bias was increased from 0 V to −5 V, the capacitance of the 200 μm diameter diode reduced from (92.50 ± 0.01) pF to (84.05 ± 0.01) pF, and the capacitance of 400 μm diameter diode reduced from (134.98 ± 0.02) pF to (121.66 ± 0.02) pF. The capacitance of the 200 μm diameter diode increased from (92.50 ± 0.01) pF at 0 V to (92.83 ± 0.01) pF at +0.3 V applied forward bias and then reduced to (92.33 ± 0.02) pF at +1.5 V applied forward bias, as shown in Fig. 1a. The peak in device capacitance when it was operated in forward bias may be attributed to increased series resistance of the diode as a result of non-ohmic behaviour of the back contact^13^,^14^. The increased series resistance results in a voltage drop across the diode which leads to a reduction in charge collection^15^. The capacitance of the 400 μm diameter diode increased from (134.98 ± 0.02) pF at 0 V to (137.443 ± 0.02) pF at +1.5 V applied forward bias, see Fig. 1b. At 0 V, the depletion widths were calculated to be (28.4 ± 1.8) nm and (78.9 ± 1.8) nm for the 200 μm and 400 μm diameter diodes, respectively. At −5 V applied reverse bias, the depletion widths were calculated to be (31.3 ± 1.8) nm and (86.4 ± 1.8) nm for the 200 μm and 400 μm diodes, respectively. Hence, the diodes’ epilayers were not fully depleted.

To electrically characterise the detectors and compute their Schottky barrier heights, dark current measurements as functions of applied bias were made at room temperature (297 K) in an environment of laboratory air. The dark current measurements were made from 0 V to −5 V reverse bias in −0.05 V decrements and 0 V to +1.5 V forward bias in 0.05 V increments. The current was measured at the front contact after allowing a period of 10 s for the current to stabilise after each voltage step. The bias was applied and the current measured using a computer controlled Keithley 6487 picoammeter/voltage source.

**Figure 2:**
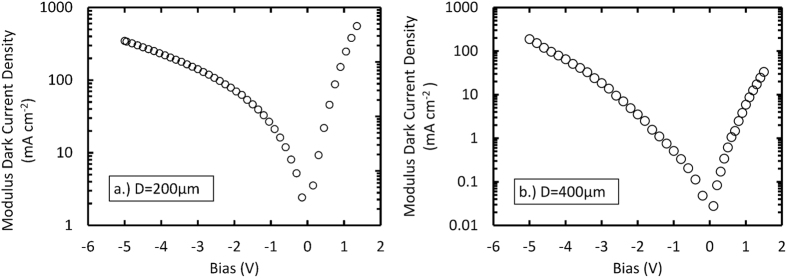
Dark current density as a function of the applied bias; (**a**) 200 μm diameter, 6 μm epilayer and (**b**) 400 μm diameter, 0.5 μm epilayer.

The dark current densities as functions of applied bias for both diodes are presented in Fig. 2. There was variation in dark current density between the two diodes; at −5 V reverse bias, the 200 μm diameter diode had a larger dark current density, (347.2 ± 0.4) mA cm^−2^, compared with the 400 μm diameter diode, (189.0 ± 0.2) mA cm^−2^.

The Schottky barrier heights at 0 V for the 200 μm diode, *φ*_*0B1*_ = (0.52 ± 0.07) eV, and the 400 μm diode, *φ*_*0B2*_ = (0.63 ± 0.09) eV, at room temperature were obtained using the relationship between the saturation current (*I*_*s*_), temperature (*T*) and *φ*_*0B*_, given by,


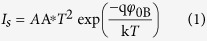


where, *A* is the area of the diode, A* is the Richardson constant (2.64 × 10^5^ A K^−2^ m^2^) [18], and k is the Boltzmann constant. The calculated barrier heights, *φ*_*0B1*_ and *φ*_*0B2*,_ are comparable to those previously reported for a Ni/Au Schottky contact on n type hexagonal GaN, (0.560 ± 0.004) eV^16^.

The two diodes were characterised for their temporal response to illumination with X-rays, while operated at +0.5 V forward bias and −0.5 V reverse bias. The X-ray source was an X-ray tube with a molybdenum cathode, operated at 35 kV and a current of 1 mA. No monochromator was used so that the X-ray spectrum also contained bremsstrahlung radiation (energy ≤ 35 keV) as well as the characteristic emission lines corresponding to the molybdenum cathode (Kα = 17.4 keV; Kβ = 19.6 keV). In turn, each diode was placed in a custom dark box with a 4 μm thick Al foil X-ray window. Using a computer controlled Keithley 6487 picoammeter/voltage source, the dark current was measured at the front contact of the diode every 20 s for a period of 200 s before the X-ray tube was switched on. Subsequently, the X-ray tube was switched on and the diode’s current was measured every 20 s for another period of 200 s before the X-ray tube was switched off. The cycle was then immediately repeated twice more. The X-ray tube was pre-warmed such that the turn on and off stabilisation times for output of the tube were <5.0 s and <2.6 s, respectively, these were significantly less than measurement interval (20 s) of the current measurements as a function of time.

**Figure 3:**
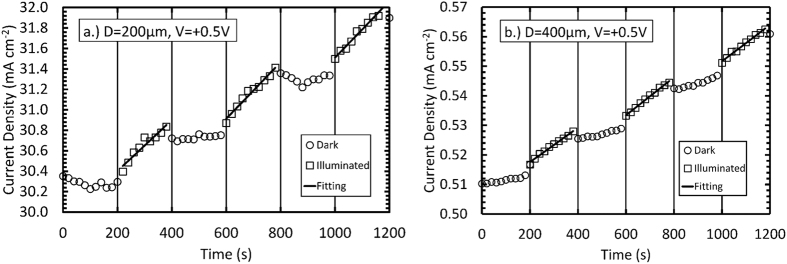
The dark current density (open circles), illuminated current density (open squares) and the linear least square fitting of the illuminated current density (black lines) at +0.5 V forward bias as a function of time for (**a**) 200 μm diameter, 6 μm epilayer and (**b**) 400 μm diameter, 0.5 μm epilayer.

**Figure 4:**
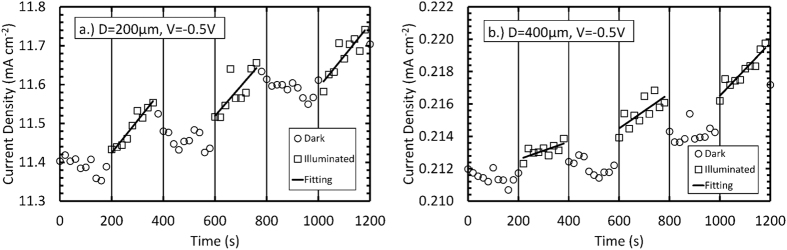
The dark current density (open circles), illuminated current density (open squares) and the linear least square fitting of the illuminated current density (black lines) at −0.5 V reverse bias as a function of time for (**a**) 200 μm diameter, 6 μm epilayer and (**b**) 400 μm diameter, 0.5 μm epilayer.

Figures 3 and 4 show a fast turn-on transient, with an increase in current seen immediately. This is followed by a slow increase in measured current over the 200 seconds illumination period. The latter region is highlighted by linear least squares fitting of the illuminated current data points. Upon turning off the X-ray tube, a fast turn-off transient was observed after which the current remained approximately constant. These results provided clear experimental evidence that the cubic-GaN Schottky diodes detected X-ray radiation.

Fast turn-on/turn-off transients have been attributed to a photovoltaic component of the current which is generated in the depletion region of the diodes^2^. Similar fast transient times with X-ray illumination of hexagonal GaN p-i-n diodes have been previously reported by Yao *et al.*^10^. When operating the presently reported diodes at +0.5 V forward bias and −0.5 V reverse bias the current measured through each diode did not return to the initial dark current value within 200 s, once the X-ray tube was switched off. In contrast to the presently reported results, in the hexagonal GaN diodes investigated by Duboz *et al.*^2^, the ramp up in detected photocurrent when the X-ray tube (photon energies up to 40 keV) was turned on was slower (100 s) than the decay in photocurrent at the cessation of illumination (1 s). Duboz *et al.* attributed these turn-on and turn-off timings to photoconductive contributions to the measured current and activation of traps in their detectors by the incident X-ray photons, respectively^2^. The increased dark current measured when operating the presently reported diodes at +0.5 V forward bias and −0.5 V reverse bias after switching off the X-ray beam could be attributed to a decrease in resistance of the material as a result of additional charge carriers created by the incident X-ray photons. After illumination ceased, the dark currents of the diodes slowly (10 minutes) returned to their original values.

A greater response was expected to be seen in the 400 μm diameter diode due to its larger depletion width (86.4 ± 1.8) nm in comparison to the 200 μm diameter diode (31.26 ± 1.8) nm. Using the Beer-Lambert law and assuming the active region of the detectors was the depletion layer and identical charge collection efficiencies in each device, the detection efficiency of the 400 μm diameter diode was expected to be 2.7 ± 0.1 that of the 200 μm diameter diode. However, when operated at −0.5 V reverse bias, the 200 μm diameter diode showed a larger increase in current density during each illumination than the 400 μm diameter diode; the mean gradients determined by linear least square fitting were (221 ± 83) nA cm^−2^ s^−1^ and (40 ± 21) nA cm^−2^ s^−1^ for the 200 μm and 400 μm diameter diodes, respectively. The greatest increase, measured from 1000 s to 1180 s, was (159.4 ± 20.0) μA cm^−2^ for the 200 μm diameter diode compared with (3.55 ± 0.58) μA cm^−2^ for the 400 μm diameter diode. The results may be explained by the collection of charge created outside of the diodes’ depletion regions by photons absorbed in those locations; the 200 μm diameter diode had a much thicker epilayer (6 μm) than the 400 μm diameter diode (0.5 μm).

When operated at +0.5 V forward bias, the 200 μm diameter diode also showed a larger increase in current density during each illumination period than the 400 μm diameter diode; the mean gradients determined by linear least square fitting were (272 ± 37) nA cm^−2^ s^−1^ and (91 ± 57) nA cm^−2^ s^−1^ for the 200 μm and 400 μm diameter diodes, respectively. The greatest increase was seen from 600 s to 780 s: (543 ± 50) μA cm^−2^ for the 200 μm diameter diode compared with (11.0 ± 1.3) μA cm^−2^ for the 400 μm diameter diode. The mean gradients determined by linear least square fitting show that a greater response to X-ray illumination was seen when operating the diodes at +0.5 V forward bias compared to −0.5 V reverse bias. This could be due to an increase in measured current due to traps being filled when operating the diodes in forward bias, and so additional charge carriers created by the X-ray photons were less likely to become trapped^2^,^10^,^11^.

In conclusion, X-ray detection has been demonstrated with c-GaN Schottky diodes at room temperature. The diodes had front Schottky contacts with barrier heights of *φ*_*0B1*_ = (0.52 ± 0.07) eV and *φ*_*0B2*_ = (0.63 ± 0.09) eV for the 200 μm and 400 μm diameter diodes, respectively. At an applied reverse bias of −5 V, the diodes had dark current densities of (347.2 ± 0.4) mA cm^−2^ and (189.0 ± 0.2) mA cm^−2^. Increases in current density as functions of time were measured when the diodes were illuminated with X-ray photons of energy up to 35 keV. The 200 μm diameter diode showed a larger increase in current density when illuminated in comparison to the 400 μm diameter diode, despite the 400 μm diameter diode’s larger depletion width. This may be attributed to collection of charge created by X-ray photons absorbed outside of the depletion region.

## Additional Information

**How to cite this article**: Gohil, T. *et al.* X-ray detection with zinc-blende (cubic) GaN Schottky diodes. *Sci. Rep.* **6**, 29535; doi: 10.1038/srep29535 (2016).
